# Mortality and Epidemiological Changes in Proximal Hip Fractures in the Course of a Pandemic

**DOI:** 10.3390/jcm11071963

**Published:** 2022-04-01

**Authors:** Domenik Popp, Arastoo Nia, Sara Silvaieh, Cornelia Diendorfer, Lukas Schmoelz, Georg Thalmann, Stefan Frank, Kevin Döring, Stefan Hajdu, Harald K. Widhalm

**Affiliations:** 1 Clinical Division of Traumatology, Department of Orthopedics and Traumatology, Medical University of Vienna, 1090 Vienna, Austria; domenik.popp@meduniwien.ac.at (D.P.); arastoo.nia@meduniwien.ac.at (A.N.); cornelia.diendorfer@gmail.com (C.D.); lukas.schmoelz@meduniwien.ac.at (L.S.); georgthalmann@hotmail.com (G.T.); s.frank@live.at (S.F.); kevin.doering@meduniwien.ac.at (K.D.); stefan.hajdu@meduniwien.ac.at (S.H.); 2 Department of Neurology, Medical University of Vienna, 1090 Vienna, Austria; sara.silvaieh@meduniwien.ac.at

**Keywords:** COVID-19, pandemic, hip fracture, mortality, epidemiological, new normal

## Abstract

Background: Coronavirus disease 2019 (COVID-19) has had an immense impact on the treatment protocols of orthopedic and trauma departments, yet its specific effect on mortality in patients with hip fractures due to possible surgical delays is still unclear. The purpose of this paper was to investigate whether the COVID-19 pandemic worsened the mortality rate of hip fracture patients. Patients and methods: This study comprised 175 prospectively included patients who (1) suffered from hip fractures, (2) presented during the Austrian state of emergency period from 15 March 2020 to 30 May 2021, and (3) were admitted to a level I trauma center. This cohort was compared with a retrospective control group of 339 patients admitted for hip fractures during the same timeframe in 2017, 2018, and 2019. Results: An admission reduction of 22% in the COVID period compared with the pre-COVID period was evident (*p* = 0.018). The 30-day mortality rate was 14.67% (pre-COVID) compared with 15.18% (*p* = 0.381). No differences in surgical complication rates or relationships between comorbidity burden and survival were observed. There were no significant changes in demographic variables, except for admission rate, gender (*p* = 0.013), and place of accident (*p* = 0.049). Conclusion: Surgeons should be reassured to take COVID-19 precautions, as this study did not show higher perioperative mortality due to COVID-19 measures. Under the current circumstances, with possibly reduced surgical and hospital bed capacities, it is expected that hip fractures may continue to require a high degree of resources.

## 1. Introduction

The exponential spread of coronavirus disease (COVID-19) has posed one of the most substantial challenges to health care systems in recent decades [1,2], altering the needs and provision of health care worldwide and necessitating a massive reallocation of workforce and resources to prevent significant shortages (e.g., personnel on sick leave or quarantine, masks, and disinfectants).

Although the initial COVID-19 pandemic had only a moderate overall impact on the Austrian health care system, the orthopedic and trauma departments were severely affected in terms of surgical and hospital bed capacities. Furthermore, polymerase chain reaction (PCR) test results had to be awaited before treatment in patients with acute surgical indications. Non-deferrable surgeries were performed without PCR test results under a specific hygiene protocol [1]. Reduced staff and unpredictable trauma events forced hospitals to perform a balancing act between staff protection and patient care. Epidemiological evidence indicated a reduction in major trauma and activity-related trauma during this time, though the incidence of fragility fractures remained unchanged [2,3,4].

Elderly patients have suffered from this crisis in two ways, as they not only have a higher risk of severe COVID-19 progression [5,6,7], but also may be subject to delays in operative treatment, which may worsen outcomes [7,8,9].

The specific impact of the COVID-19 pandemic on patients with hip fractures as well as its effects on mortality and morbidity are yet to be determined, with only a small case series as a scientific source [10].

The primary purpose of this study was thus to investigate changes in the epidemiology and survival rate of hip fracture patients admitted to a level I trauma unit in the shadow of a pandemic.

## 2. Materials and Methods

A prospective cohort study with a retrospective control group was conducted. The study collected data on all patients (1) admitted to the Department of Orthopedics and Trauma Surgery, (2) diagnosed with a hip fracture (OTA/AO 31, 32.1), and (3) admitted during the study period. Five distinct periods were examined: the 80-day Austrian state of emergency (16 March 2020 to 30 May 2020), the same timeframe in the following year, and the same timeframe in 2017, 2018, and 2019. The years 2017, 2018, and 2019 are hereafter referred to as “pre-COVID,” and the years during the pandemic (2020 and 2021) are referred to as “COVID.” The prospectively collected COVID group was compared with the retrospective pre-COVID group. Patients presenting with periprosthetic femoral fractures, fractures due to polytrauma, and pathological fractures were excluded. The manuscript was prepared according to the STROBE guidelines for reporting observational studies.

### 2.1. Variables

The principal parameters investigated in this study included demographic variables such as age, gender, body mass index (BMI), comorbidities, type of anesthesia, choice of discharge facility, American Society of Anesthesiologists (ASA) grade, fracture type, place of accident, and postoperative complications. Postoperative complications included anemia, infection, pneumonia, delirium, thrombosis, and cardiopulmonary affections. Anemia was defined as a hemoglobin level <9 g/dL or the need for blood transfusions. Infections and pneumonia were considered distinct complications due to the pandemic. Infection was defined as local inflammation, elevated C-reactive protein levels, and fever without any signs of pneumonia on the X-rays. Delirium was defined as a postoperative change from the observed preoperative baseline mental functioning. Cardiopulmonary affections encompassed therapy-resistant systolic blood pressure >160 mmHg, postoperative chest pain with abnormal electrocardiographic alterations, and dyspnea.

Time from accident to surgery, operating time, and type of treatment were also retrieved. Postoperative outcomes were described as 30-day mortality, and causes of death were derived from the registry of deaths of the Austrian Federal Institute for Statistics.

### 2.2. Statistical Analysis

Descriptive statistics, including categorical variables (absolute and relative frequencies) and continuous ones (number of observations, mean, standard deviation (SD), median, minimum and maximum), were initially calculated separately for each year. The Chi^2^-test was used for categorical variables. Metric variables were analyzed using the Kologomorov–Smirnov test when normally distributed. Otherwise, the Mann–Whitney U test was applied.

To identify potential differences between the test and control cohorts in patient characteristics, the Chi^2^-test was used for place of accident, fracture type, surgical treatment, and ASA score, whereas the *t*-test/Mann–Whitney U test was applied for ordinal variables such as patient age. Either a two-sided *t*-test (if assumptions were not violated) or a two-sided U-test to compare hip care (defined as the duration between accident and surgery and type of surgery) between COVID and pre-COVID periods was performed. To analyze overall survival, univariate Cox regressions were conducted for interactions between the time periods and different risk factors (age, hip care, and postoperative complications).

A *p*-value of less than 0.05 was considered statistically significant.

In the case of an insignificant interaction term, only the results of the Cox model with main effects were presented. Afterwards, a multivariate Cox regression was conducted with significant variables from the univariate regressions. Statistical analyses were performed utilizing SPSS software for Mac (version 21, IBM, SPSS, Armonk, NY, USA).

## 3. Results

### 3.1. Pre-COVID vs. COVID

**Figure 1:**
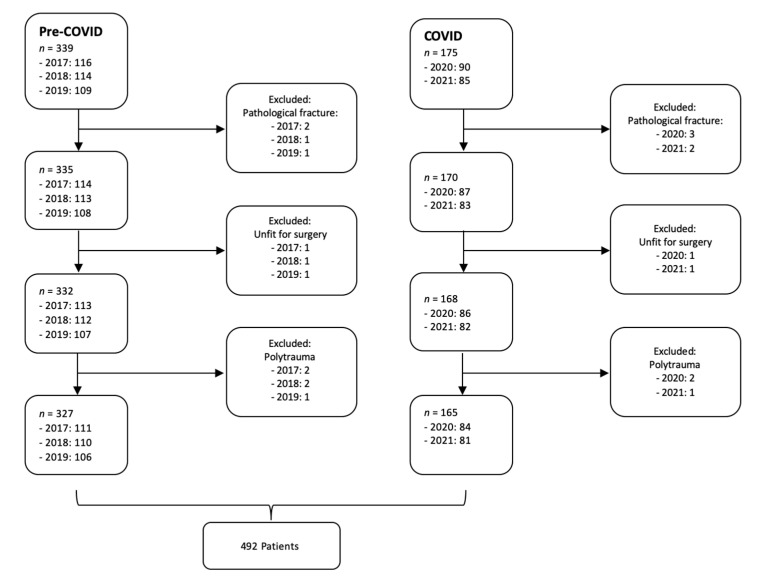
Study flow diagram.

In total, 514 patients were identified in the described time periods. The 2017, 2018, and 2019 groups showed no significant between-group differences and were therefore merged into one pre-COVID group. The pre-COVID group comprised 339 patients, using data sets for 116 patients in 2017, 114 patients in 2018, and 109 patients in 2019. The COVID group consisted of 175 patients, using data from 90 patients in 2020 and 85 patients in 2021 (Figure 1). This represented an overall mean reduction in admissions caused by a hip fracture of 22% (*p* = 0.018).

Twenty-two patients were excluded based on the exclusion criteria, resulting in 492 patients included in further analysis (Table 1). Five patients were not fit for surgery due to severe comorbidities with terminal illnesses and died within 48 h. Polytrauma and pathological fractures were also excluded due to an exponentially higher 30-day mortality rate. Most patients were over 60 years of age (93% pre-COVID vs. 95% COVID; *p* = 0.087). Females made up 70.35% of the pre-COVID group and 62.44% of the COVID group (*p* = 0.013). No significant differences in age distribution between the individual groups were found (*p* = 0.126). The mean age in 2017 was 80.06 years (standard deviation (SD): 12.04; range: 38–100), in 2018 was 79.96 years (SD: 11.88; range: 46–101), in 2019 was 79.84 years (SD: 11.97; range: 49–98), in 2020 was 80.13 years (SD: 11.76; range: 39–106), and in 2021 was 79.96 years (SD: 11.83; range: 43–100).

The proportions of hip fractures and surgery for hip fractures were almost similar in pre-COVID and COVID periods, as shown in Table 1. 

No significant differences were observed between groups in postoperative complication rates (Table 2). In the pre-COVID group, 130 patients (39.88%) suffered from 150 postoperative complications. In the COVID group, 64 patients (38.79%) suffered from 73 complications (*p* = 0.063). Anemia constituted the most frequent postoperative complication in both groups (70.70% pre-COVID and 68% in the COVID period; *p* = 0.058).

The 30-day mortality rate was 14.67% in the pre-COVID period and 15.18% in the COVID period (*p* = 0.381). Cox regression models were used to assess the independent association of time period and mortality risk and were adapted for ASA grade, age, gender, fracture type, and time until surgery (Table 3). Univariate Cox analyses showed that the factors of age over 80 (*p* = 0.019), male gender (*p* = 0.008), delayed surgery for more than 48 h (*p* = 0.002), and pertrochanteric fractures (*p* = 0.024) were significantly associated with 30-day mortality (Table 3). No seasonal influence (pre-COVID vs. COVID) on overall survival was detected. According to multivariate Cox regression, the main dependent variables for mortality were age and gender (Table 4). Significant changes in places of accident were observed from the pre-COVID to the COVID period. The mean percentage of outdoor injuries was reduced from 25.67% pre-COVID to 9.74% during the pandemic (*p* = 0.011). There were no significant changes in demographic data between pre-COVID and COVID periods, except for an increased number of fractures in male patients during the COVID period (29.64% pre-COVID vs. 37.56 COVID; *p* = 0.035). Age, body mass index, smoking habits, and chronic underlying medical conditions such as previous myocardial infarction, diabetes, dementia or chronic obstructive pulmonary disease were not significantly different between periods (Table 5). No changes in the choice of discharge facility were seen, equally to the constant number of cases of spinal anesthesia for surgery (*p* = 0.066).

### 3.2. Pre-COVID vs. 2020

A delay in the mean time from admission to surgery for proximal hip fractures occurred in 2020. During the 2020 COVID period, 69.05% of patients underwent surgery within 24 h and 85.71% underwent surgery within 48 h, compared with 85.89% undergoing surgery within 24 h and 95.43% undergoing surgery within 48 h during the pre-COVID period (*p* = 0.003). The median duration between accident and surgery was 6 h in the pre-COVID period and 22 h in the 2020 COVID period, with COVID-19 safety precautions (e.g., PCR results) responsible for surgical delays of approximately 24 h (*p* = 0.001).

### 3.3. Pre-COVID vs. 2021

In 2021, very few differences were seen compared with pre-COVID levels across all variables. Male gender was still more common in hip fracture patients in 2021 compared with pre-COVID (*p* = 0.044). Duration from admission to surgery increased from 6 h pre-COVID to 8 h in 2021 (*p* = 0.039). The frequency of outdoor injuries continued to be significantly lower in 2021, with a decrease in outdoor injuries of approximately 64% in 2021 compared with pre-COVID (*p* = 0.031).

### 3.4. COVID: 2020 vs. 2021

Time from admission to surgery decreased significantly in 2021 compared with 2020, showing close to pre-COVID values for rates of surgical treatment within 24 h (*p* = 0.006) and 48 h (*p* = 0.009). The median duration between accident and surgery shortened from 22 h in 2020 to 8 h in 2021 (*p* = 0.003).

A trend of returning to pre-COVID figures was observed in 2021 in place of accident, as the number of outdoor injuries increased again by 67% in 2021 compared with 2020 (*p* = 0.021).

## 4. Discussion

The COVID-19 pandemic, as an ongoing crisis, has had a vast impact on patient management and health care resources in orthopedic and trauma departments, with particular complications in acute surgical settings. As the COVID-19-specific literature on the outcomes of hip fracture patients is sparse, and investigations of pandemic effects on treatment outcomes are needed, we sought to provide data on demographic and patient management changes and their impact on short-term mortality in hip fracture patients. We found that the pandemic caused a one-day delay of surgery during its first year. In 2021, time from admission to surgery returned to almost pre-COVID levels, which is attributed to the widespread availability of faster and newer PCR test kits, reducing the time needed for a COVID-19 result from 20 h to 70 min.

Nia et al. [11] described a decrease in all patient admissions by almost 70% in 2020. Compared with other conditions, the incidence of hip fractures was less affected by the social lockdown in the COVID period. A decrease in hip fractures of only 22% was observed, which significantly increased the percentage of hip fracture surgeries of all surgeries during the COVID period. This is consistent with the literature, in which milder changes in the incidence of hip fractures during the COVID-19 pandemic relative to other trauma conditions have been reported [12].

Compared with the pre-COVID period, short-term mortality did not rise in patients with hip fractures during the state of emergency. These results support the hypothesis that the lower incidence of hip fractures was associated with the state of emergency in combination with the stay-at-home strategy, and was not due to higher mortality as a result of COVID-19. Scott et al. [13] and Ojeda-Thies et al. [14] observed a reduction in admissions for hip fractures as well. As a situation such as the state of emergency had never previously occurred, a change in social behavior and related physical activity patterns may explain these findings. Many falls occur outdoors or during normal daily activities; therefore, the lockdown may have contributed to a lower fall rate and subsequently a decreased frequency of hip fractures. Staying home during the lockdown, where people are comfortable and familiar with their surroundings, and avoiding outside activities in various weather conditions might have reduced the chances of an adverse event such as a hip fracture [15].

Some studies have suggested that the reductions in hospital admissions were associated with the fear of infection in outpatient departments [16,17]. This might be a reasonable explanation for many non-surgical diseases such as kidney failure or myocardial infarction, with fear causing people to stay home rather than to seek help [16,17]. In the case of hip fractures, however, this explanation is not suitable, as surgical treatment is inevitable and usually cannot be delayed due to the severe functional limitations and tremendous pain. An additional possible explanation for the comparatively mild reduction in hip fractures lies in the patient population. More than 90% of patients in all groups were older than 60 years, with a mean age of 80 years. This age group often suffers from severe comorbidities, such as osteoporosis, malnutrition and sarcopenia, making it virtually inevitable that a serious injury will occur from a fall or a minor accident.

Nonetheless, case series describing hip fractures and their survival rates during a global pandemic are still sparse. In general, consistent with the findings of Hershkowitz et al. [18] and Moshammer et al. [19], there was no relationship between comorbidity burden and patients’ survival. This might be due to the homogeneity of this group and the high prevalence of cardiovascular diseases, as well as the reduced nutritional and physical status [19,20,21,22].

Perioperative mortality did not change during the COVID-19 pandemic in this study and was aligned with national statistics and other published data [2,3,4,23]. Multivariate Cox regression showed that old age and male gender were the principal factors for mortality [15].

A multitude of changes in health care systems occurred due to COVID-19, including reduced operating room capacity and depleted staff. The findings of this study should aid medical professionals in their preparative decision-making processes. Hip fractures might require relatively more resources during a pandemic, as this patient population and its needs are not reduced at the same rate as other trauma conditions.

## 5. Limitations

Due to the retrospective nature of the control group, the measurement of laboratory parameters and the history of disease might be biased in the control group compared with the prospective group. A prospective control group was not possible due to the sudden onset of the pandemic. Furthermore, demographic data collected retrospectively in 2017, 2018, and 2019 and prospectively in hospital admissions in 2020 and 2021 are unlikely to impact treatment decisions, considering the short period between admission and surgery and the mandatory surgical indications in most cases.

As the study employed short follow-ups and a relatively small sample, the mortality rate might be underestimated. Restriction to a short timeframe was necessary to reflect the relatively short state-of-emergency period, which was a precondition for this study.

Selection bias cannot be ruled out, as a higher number of femoral head-preserving surgeries might have been chosen in borderline cases to reduce perioperative complications and surgical staff exposure during COVID-19 times. Although this unavoidable limitation should not be neglected, we believe that our department did not deviate from standard protocol. Furthermore, borderline cases were rare and in these cases, patient comorbidities, age, and, ultimately, surgeon’s choice were already influencers of surgical indication in non-COVID-19 times.

Due to low patient numbers, no differentiated perioperative survival analyses after different forms of hip fracture surgery could be given in this study. However, this study only aimed to give a more generalized answer to surgical implications in times of a global pandemic.

## 6. Conclusions

This study demonstrated that hip fracture patients during the pre-COVID and the COVID periods had similar comorbidity and demographic profiles with no significantly increased rates of short-term mortality. A reduction in hip fracture cases was observed during the COVID period, though the rate was not reduced to the same degree as has been reported for other trauma conditions. It is thus expected that hip fractures may continue to require a high level of resources in times of potentially scarce capacities, such as during a pandemic.

## Figures and Tables

**Table 1:** Epidemiological data—surgical and fracture characteristics.

	2017		2018		2019		2020		2021	
	*n*	%	*n*	%	*n*	%	*n*	%	*n*	%
Total	116		114		109		90		85	
Excluded	5		4		3		6		4	
Patho Fx	2	40.00	1	25.00	1	33.33	3	50.00	2	50.00
Unfit	1	20.00	1	25.00	1	33.33	1	33.33	1	25.00
Polytrauma	2	40.00	2	50.00	1	33.33	2	13.67	1	25.00
*p*-Value	0.81		0.35		0.51		-		0.49	
Surgery	111		110		106		84		81	
*p*-Value	0.006		0.009		0.012		-		0.089	
Fracture type										
Intra. n.d.	12	10.81	12	10.91	13	12.26	10	11.90	9	11.11
Intra	40	36.03	39	35.46	41	38.67	33	39.29	32	39.51
Pertrochanteric	52	46.85	54	49.09	49	46.23	40	47.61	34	41.98
Subtrochanteric	7	6.31	5	4.54	3	2.84	1	1.20	2	2.47
*p*-Value	0.081		0.099		0.123		-		0.079	
Surgery type										
HEP	33	29.73	32	29.09	35	33.01	28	33.33	32	39.51
THA	7	6.30	7	6.36	6	5.66	5	5.95	4	4.93
IM Nail	50	45.05	52	47.28	45	42.46	37	44.05	34	41.98
DHS	9	8.11	7	6.36	7	6.61	4	4.76	2	2.47
P.C.S.	12	10.81	12	10.91	13	12.26	10	11.91	9	11.11
*p*-Value	0.089		0.738		0.788		-		0.998	

Patho Fx: pathological fracture; intra. n.d.: intracapsular not dislocated; THA: total hip arthroplasty; HEP: hemiarthroplasty; IM: intramedullar; DHS: dynamic hip screw; P.C.S.: percutaneous cannulated screw.

**Table 2:** Epidemiological data—surgical and general characteristics.

	2017		2018		2019		2020		2021	
Time to Surgery	*n*	%	*n*	%	*n*	%	*n*	%	*n*	%
<24 h	95	86.36	93	84.54	92	86.79	58	69.05	68	83.95
<48 h	104	94.54	105	95.54	102	96.22	72	85.71	77	95.06
*p*-Value	0.011		0.019		0.025		-		0.009	
ASA										
1	7	6.31	5	4.54	4	3.77	5	5.95	4	4.95
2	36	32.43	37	33.64	36	33.96	26	30.95	27	33.33
3	64	57.66	65	59.09	64	60.38	51	60.70	48	59.26
4	4	3.60	3	2.73	2	1.89	2	2.4	2	2.46
*p*-Value	0.141		0.085		0.167		-		0.762	
Place of accident										
Home	79	71.17	80	72.73	79	74.53	77	91.67	71	87.65
Outdoor	30	27.03	28	25.45	26	24.53	6	7.14	10	12.34
Hospital	1	0.90	2	1.82	1	0.94	1	1.19	0	0.00
*p*-Value	0.037		0.028		0.044		-		0.021	
Complications	53		50		47		38		35	
Anemia	37	69.81	35	70.00	34	72.30	27	71.05	24	68.57
Infection	2	3.77	2	4.00	1	2.13	1	2.63	1	2.86
Delirium	4	7.56	4	8.00	3	6.38	3	7.89	3	8.57
Cardiopulm affect	3	5.66	3	6.00	2	4.23	2	5.26	2	5.72
Thrombosis	2	3.77	3	6.00	2	4.23	1	2.63	2	5.72
Pneumonia	5	9.43	3	6.00	5	10.63	4	10.52	3	8.56
*p*-Value	0.550		0.682		0.420		-		0.839	
Mortality	17	15.31	16	14.55	15	14.15	13	15.55	12	14.81
*p*-Value	0.068		0.073		0.103		-		0.097	

ASA: American Association of Anesthesiology; Cardiopulm affect: Cardiopulmonary affections.

**Table 3:** Cox univariate regressions: overall survival is a dependent variable.

Risk Score	HR	(95%-CI)	*p*-Value
Age	1.091	(1.015–1.174)	0.019
Period	0.393	(0.079–1.961)	0.255
Dur ad op	1.674	(1.204–1.961)	0.002
Pertroch Fx	1.566	(1.108–1.883)	0.024
Gender	1.123	(1.006–1.169)	0.008
ASA	0.404	(0.098–1.889)	0.079

Dur ad op: duration between admission and surgery; Pertroch Fx: pertrochanteric fracture; ASA: American Association of Anesthesiology. Note: HR—hazard ratio, CI—confidence interval.

**Table 4:** Multivariate Cox regression for OS.

	HR	CI Lower Bound	CI Upper Bound	*p*-Value
Age	1.098	1.001	1.205	0.049
Dur ad op	1.405	0.916	2.153	0.119
Pertroch Fx	1.496	0.988	2.008	0.138
Gender	1.081	1.014	1.198	0.035

Dur ad op: duration between admission and surgery; Pertroch Fx: pertrochanteric. Note: HR—hazard ratio, CI—confidence interval, OS—overall survival.

**Table 5:** Epidemiological and demographic characteristics.

	2017		2018		2019		2020		2021	
	*n*	%	*n*	%	*n*	%	*n*	%	*n*	%
Gender	111		110		106		84		81	
Male	33	29.73	34	30.90	30	28.30	32	38.09	30	37.03
Female	78	70.27	76	69.10	76	71.70	52	61.91	51	62.97
*p*-Value	0.036		0.038		0.041		-		0.066	
BMI	23.78	(3.9)	23.81	(4.1)	23.19	(3.9)	2369	(4.0)	23.57	(3.8)
*p*-Value	0.19		0.089		0.059		-		0.27	
Tobacco Smoker										
Current	3	2.70	2	1.82	3	2.83	2	2.38	2	2.47
Former	28	25.23	26	23.64	24	22.64	21	25.00	20	24.69
Never	69	62.16	67	60.91	65	61.32	52	61.90	50	61.73
Unknown	11	9.91	15	13.63	14	13.21	9	10.72	9	11.11
*p*-Value	0.51		0.29		0.19		-		0.36	
Hx of MCI	8	7.20	9	8.18	8	7.55	6	7.14	5	6.17
*p*-Value	0.48		0.94		0.33		-		0.24	
Cong Heart Fail	6	5.40	6	5.45	7	6.60	5	5.96	5	6.17
*p*-Value	0.67		0.56		0.21		-		0.34	
PVD	6	5.40	7	6.36	6	5.66	5	5.96	4	4.94
*p*-Value	0.77		0.45		0.38		-		0.42	
COPD	8	7.20	7	6.36	7	6.60	6	7.14	6	7.41
*p*-Value	0.63		0.58		0.41		-		0.71	
Dementia	18	16.22	18	16.36	17	16.04	14	16.67	13	16.05
*p*-Value	0.81		0.33		0.91		-		0.65	
Hx of Stroke	4	3.60	5	4.55	4	3.77	3	3.57	4	4.94
*p*-Value	0.44		0.089		0.099		-		0.21	
DMII	14	12.61	13	11.82	14	13.20	11	13.10	9	11.11
*p*-Value	0.19		0.29		0.35		-		0.63	
CKD	6	5.40	6	5.45	7	6.60	5	5.96	4	4.94
*p*-Value	0.27		0.55		0.87		-		0.91	
Liver Disease	2	1.82	1	0.91	1	0.94	1	1.19	1	1.23
*p*-Value	0.71		0.76		0.52		-		0.32	
Discharge Facility										
Home	52	46.85	50	45.45	48	45.28	38	45.24	37	45.68
Retirement Home	44	39.64	45	40.92	44	41.51	35	41.67	33	40.74
P.T.C.	13	11.71	12	10.91	12	11.32	9	10.71	9	11.11
Hospice	2	1.80	3	2.72	2	1.89	2	2.38	2	2.47
*p*-Value	0.53		0.62		0.78		-		0.84	
Anesthesia Type										
Spinal	76	68.47	76	69.09	73	68.87	58	69.05	55	67.90
General	35	31.53	34	30.91	33	31.13	26	30.95	26	32.10
*p*-Value	0.098		0.12		0.067		-		0.11	

Hx: history; MCI: myocardial infarction; BMI: body mass index; Cong Heart Fail: congestive heart failure; PVD: peripheral vascular disease; COPD: chronic obstructive pulmonary disease; DMII: diabetes mellitus type 2; CKD: chronic kidney disease; P.T.C.: physical therapy centre; categorical variables are reported in *n* (% of total), continuous parametric variables are reported as mean (SD).

## Data Availability

Not applicable.
